# Antioxidant Activity of Citrus Limonoids and Investigation of Their Virucidal Potential against SARS-CoV-2 in Cellular Models

**DOI:** 10.3390/antiox10111794

**Published:** 2021-11-10

**Authors:** Fabio Magurano, Marzia Sucameli, Pasquale Picone, Matteo Micucci, Melissa Baggieri, Antonella Marchi, Paola Bucci, Silvia Gioacchini, Giorgia Catinella, Gigliola Borgonovo, Sabrina Dallavalle, Domenico Nuzzo, Andrea Pinto

**Affiliations:** 1 Department of Infectious Diseases, Istituto Superiore di Sanità, Viale Regina Elena, 299, 00161 Rome, Italy; fabio.magurano@iss.it (F.M.); melissa.baggieri@iss.it (M.B.); antonella.marchi@iss.it (A.M.); paola.bucci@iss.it (P.B.); silvia.gioacchini@guest.iss.it (S.G.); 2 Department of Internal Medicine and Medical Specialties (DIMI), University of Genoa, V.le Benedetto XV 6, 16132 Genoa, Italy; sucamelimarzia@virgilio.it; 3 Istituto per la Ricerca e l’Innovazione Biomedica (IRIB), CNR, Via Ugo La Malfa 153, 90146 Palermo, Italy; pasquale.picone@irib.cnr.it; 4 UniCamillus—Saint Camillus International University of Health Sciences, Via di Sant’Alessandro, 800131 Rome, Italy; matteo.micucci2@unibo.it; 5 Nutraceutical Lab, Department of Pharmacy and Biotechnology, Alma Mater Studiorum, University of Bologna, Via Belmeloro 6, 40126 Bologna, Italy; 6 Department of Food, Environmental and Nutritional Sciences, Università degli Studi di Milano, Via Celoria 2, 20133 Milan, Italy; giorgia.catinella@unimi.it (G.C.); gigliola.borgonovo@unimi.it (G.B.); sabrina.dallavalle@unimi.it (S.D.)

**Keywords:** COVID-19, SARS-CoV2, grapefruit seeds, limonoids, antioxidant activity

## Abstract

The COVID-19 pandemic represents an unprecedented global emergency. Despite all efforts, COVID-19 remains a threat to public health, due to the complexity of mass vaccination programs, the lack of effective drugs, and the emergence of new variants. A link has recently been found between the risk of developing a severe COVID-19 infection and a high level of oxidative stress. In this context, we have focused our attention on natural compounds with the aim of finding molecules capable of acting through a dual virucidal–antioxidant mechanism. In particular, we studied the potential of grapefruit seed extracts (GSE) and their main components, belonging to the class of limonoids. Using chemical and biological approaches including isolation and purification of GSE, antioxidant and virucidal assays, we have shown that grapefruit seed constituents, belonging to the class of limonoids, are endowed with remarkable virucidal, antioxidant and mitoprotective activity.

## 1. Introduction

Coronaviruses (CoVs) are large, positive-stranded, enveloped RNA viruses that generally cause enteric and respiratory diseases in humans and in animals. Most human CoVs have recently attracted global attention to their lethal potential and great infectious capacity. A highly pathogenic CoV, called SARS-CoV-2, dramatically emerged in December 2019 in Wuhan, China and has rapidly spread around the world during the COVID-19 (CoronaVirus Disease 19) pandemic. SARS-CoV-2 infection and the destruction of lung cells triggers a local immune response, recruiting macrophages and monocytes to respond to the infection. The increased release of cytokines produces an excessive amount of reactive oxygen species (ROS) in various tissues. Viral infections are often associated with acute oxidative stress, influencing the pathogenesis of diseases as well as the inflammatory response in several organs and tissues, such as the brain, endothelium and gastrointestinal tract [1,2,3,4]. There are several common links between the risk of developing a severe COVID-19 infection and a high level of oxidative stress. In fact, a number of major risk factors related to COVID-19 severity and mortality have been identified: older age, hyperglycemia, hypertension, and obesity [5], factors strongly associated with increased oxidative stress [6,7]. In addition, several in vitro and in vivo studies have shown that some viruses alter the redox balance of a cell [8,9,10]. Furthermore, the prolonged clinical consequences linked to COVID-19 have recently been analyzed [11]. Controlling the oxidative stress response may be as important as targeting the virus. Therapies inhibiting viral infection (antiviral) and regulation of dysfunctional immune responses and oxidative stress may synergize to block pathologies at multiple steps. It can be assumed that the high level of oxidative stress aggravates the prognosis of COVID-19. Antioxidants prevent or slow down the damage to cells caused by ROS. The neutralizing activity of radical molecules by antioxidants is achieved through their scavenging power by blocking chain reactions, peroxide decomposition, and the induction of antioxidant enzymes. Interest has grown in the idea that oxidative stresses underlie the etiology of numerous diseases. The oxidative insult can arise through increased production of ROS and/or can be due to a lack of antioxidant defenses, and this can further worsen COVID-19. Free radicals are common byproducts of aerobic cellular metabolism that the body can normally handle. In the presence of a secondary condition, such as SARS-CoV-2 infection, the excessive level of radicals can contribute to disease progression and pathogenesis. In this context, the supplementation of antioxidants could reduce complications related to infection [12].

Diverse vaccines have been developed to treat COVID-19. According to the World Health Organization (WHO), more than 108 vaccine candidates have been under clinical evaluation [13]. However, a certain portion of the population cannot benefit from the vaccine. On the other hand, the real efficacy and safety of current therapeutic approaches will need further scrutiny when an adequate amount of clinical data becomes available. Updated reports about the COVID-19 disease management have targeted its structure, pathology, and mechanism in order to obtain the best treatment. The Food and Drug Administration (FDA) has created a special emergency program for possible coronavirus therapies, known as the Coronavirus Treatment Acceleration Program (CTAP). The program uses every available method to move new treatments to patients as quickly as possible, while at the same time finding out whether they are helpful or harmful. Small molecules acting on a specific viral target may represent potential tools to contrast COVID-19 and future viral threats. Nature-derived bioactives (NDBs) have been used throughout history as traditional medicines, and many clinically used drugs have been inspired by natural products, which constitute a broad biodiversity of molecules naturally selected over millennia of evolution. Several NDBs are able to counteract oxidative stress [1,2,3,4,5,6,7], thus also presenting an antiviral effect [14,15,16,17,18]. Recently, Prasansuklab and coworkers reviewed potential antiviral natural compounds with multiple targets related to coronaviruses. This research opened future prospective about some classes of natural compounds [14]. Most of the active molecules showed the inhibition of the enzyme Mpro in millimolar concentrations [15,16,17,18].

Grapefruit seed extract (GSE) is well known for its antimicrobial properties. In a recent study, the effect of GSE has been evaluated on avian influenza virus (AIV), Newcastle disease virus (NDV) and infectious and bursal disease virus (IBDV). GSE has showed virucidal activity against AIV and NDV, which are enveloped viruses. However, it was not able to show virucidal activity against the nonenveloped virus IBDV [19,20]. Notwithstanding this promising evidence, to date, there are a few studies addressing the antiviral activity of GSE, and, to the best of our knowledge, no reports on the activity of GSE against SARS-CoV-2. Moreover, recent studies have suggested that GSE has antioxidant capacity [21,22]. Lipiński and collaborators confirmed that GSE could exert antioxidant activities by improving serum SOD, GSH-PX, T-AOC and CAT and by decreasing serum MDA. This means that GSE can be an effective antioxidant, which could reduce reactive free radicals and oxidative stress by activating the antioxidant enzyme system [23]. Furthermore, GSE has a high growth inhibition effect against Gram-negative bacteria, such as *Pseudomonas aeruginosa* and *Escherichia coli*, as well as Gram-positive bacteria, such as *Staphylococcus* spp. and *Enterococcus* spp. [24,25,26]. Due to its high bactericidal effect and its safety, the extract is widely used in the food industry to retard or reduce bacterial growth, thus extending the shelf-life of food [17]. Despite the vast literature, the mechanisms of action of GSE are only partly understood, and further investigations are warranted in order to establish the molecular targets responsible for the antioxidant and antiviral proprieties. Intrigued by the possibility of having a safe and easily accessible natural extract in the arsenal of weapons against SARS-CoV-2, in the present work we aimed to evaluate the ability of extracts of grapefruit seeds to directly counteract SARS-CoV-2 infection using a two-pronged approach, which involves both virucidal and antioxidant activities.

Starting from grapefruit seeds, we have generated a small collection of extracts, enriched fractions, and single molecules, which have been fully chemically characterized. All fractions and single components were evaluated for their virucidal and antioxidant activities. The collected results evidence that the most active constituents are terpenoids belonging to the family of limonoids and flavonoid glycosides.

## 2. Materials and Methods

### 2.1. Chemicals and Equipment

All solvents (acetonitrile, hexane, ethyl acetate, dichloromethane, ethanol, *N*-heptane, *N*-butanol, acetone, CDCl_3_, CD_3_OD) were obtained from Merck Life Science (Milan, Italy). Isolation and purification of the compounds were performed by flash column chromatography on silica gel 60 (230–400 mesh). Analytical TLC was conducted on TLC plates (silica gel 60 F254, aluminum foil). Compounds on TLC plates were detected under UV light at 254 and 365 nm or were revealed by solution of anisaldehyde (2%) and H_2_SO_4_ (2%) in EtOH. NMR spectra were recorded on 600 MHz spectrometer Bruker Avance. High-Resolution Electrospray Mass Spectra were acquired with Q-TOF Synapt G2 Si (WATERS), available at COSPECT Unitech Platform (Milan, Italy). HPLC analyses were performed with a liquid Chromatograph Varian ProStar (Varian Inc., Palo Alto, CA, USA), equipped with a ternary pump with a UV–Vis detector Varian Model 345. An RP 18 (Hypersil ODS, Thermo, 5 μm 300 × 4 mm i.d.) was used. All the samples were dissolved in ACN:H_2_O 3:7 and filtered with 0.45 μm nylon filters. The elutions were performed with ACN and Water milliQ, flow 1 mL/min, λ 210 and 280 nm. The condition used were the following: t 0–15 min from ACN 10% to 60%; t 15–40 min at ACN 60%. Preparative HPLC was performed with a C-18 Ascentis, Supelco (21.2 i.d. × 250 mm, 5 μm), fitted to a 1525 Extended Flow Binary HPLC pump and a Waters 2489 UV–Vis detector (both from Waters, Milan, Italy). The separation was performed in gradient condition: t 0–30 min from ACN 10% to 30%, at a flow rate of 15 mL/min, monitoring the eluate at 210 nm.

### 2.2. Plant Material and Extractions

**Figure 1:**
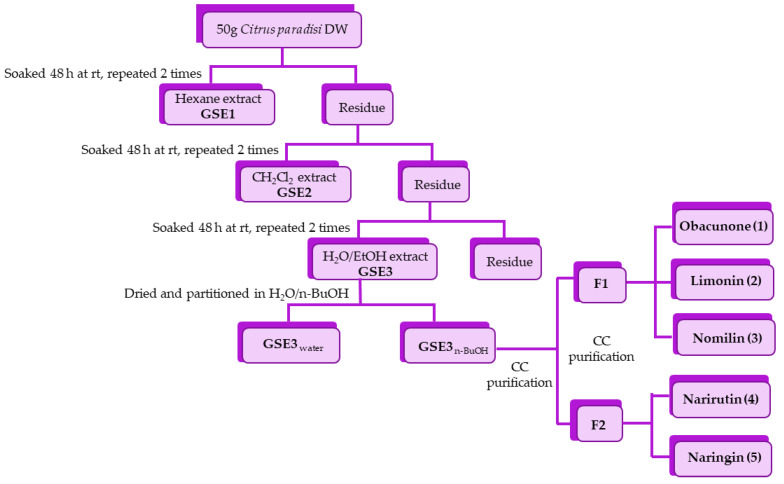
Schematic representation of the extraction–purification process from *Citrus paradise* seeds.

**Figure 2:**
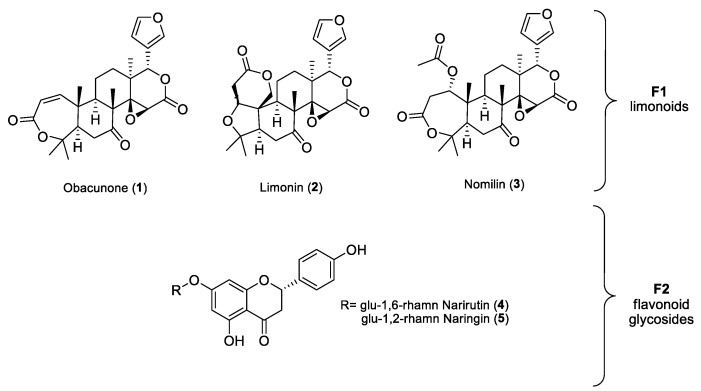
Structures of purified limonoids and flavonoid glycosides. Fraction **F1**: obacunone (**1**), limonin (**2**) and nomilin (**3**); fraction **F2**: narirutin (**4**) naringin (**5**).

*Citrus paradisi* seeds were collected during the summer 2020 in Cefalù, Contrada Parandola, Sicily. Seeds were dried in an oven (RGV Digital Dried) at 40 °C until constant weight. Dried seeds were ground, and the powder (20 g) was sequentially extracted in the dark for 48 h at room temperature with hexane (2 × 100 mL) to remove the fatty matter, yielding **GSE1**, dichloromethane (2 × 100 mL) to obtain extract **GSE2**, and ethanol/water 1:1 (100 mL) to obtain extract **GSE3**. All the extracts were filtered and evaporated under reduced pressure to afford **GSE1** (10.0 g), **GSE2** (0.8 g), and **GSE3** (5.4 g) and analyzed by HPLC (Figure 1 and Figure 2). Preliminary virucidal activity evaluation evidenced **GSE3** as the most active fraction (see Section 4).

### 2.3. Isolation and Purification

The hydroalcoholic extract (**GSE3**, 3.1 g) was dissolved in 100 mL of H_2_O and extracted with n-BuOH (3 × 30 mL). The two phases in turn were dried under reduced pressure to create fractions **GSE3_water_** (2.4 g) and **GSE3_BuOH_** (0.62 g), and were analyzed by HPLC. **GSE3_BuOH_** was purified by silica-gel column chromatography using a mixture of EtOAc:acetone:H_2_O:HCOOH (8:1:0.5:0.5 and 7:2:0.5:0.5) as eluent. Two main fractions were isolated, **F1** containing a mixture of three limonoids and **F2** containing a mixture of two flavonoid glycosides. Fraction **F1** (45 mg) was subjected to silica-gel column chromatography with hexane:EtOAc (3:7) to yield three fractions: obacunone (**1**) (4 mg), limonin (**2**) (30 mg), nomilin (**3**) (7 mg). Fraction **F2** (35 mg) was purified by preparative HPLC to yield narirutin (**4**) (4.8 mg, rt 17.30 min) and naringin (**5**) (2.1 mg, rt 18.15 min). The structures of isolated limonoids and flavonoid glycosides were confirmed by 1D and 2D NMR and MS experiments. Structural characterization of compounds is provided in Supporting Materials.

### 2.4. Investigation on Extraction Procedure 

Once identified the most active compounds, alternative extraction protocols were investigated: (1) ethanol extraction (EtE), (2) acetone/ethyl acetate extraction (AAE) and (3) triphasic extraction (TE). For ethanol extraction (EtE), grapefruit seed powder (3 g) was stirred with EtOH (10 mL × 2) at rt in the dark for 48 h. The extracts were filtered and evaporated under reduced pressure. In the acetone/ethyl acetate extraction (AAE), grapefruit seed powder (3 g) was extracted with a mixture 1:1 (10 mL × 2) at rt in the dark for 48 h. The extracts were filtered and evaporated under reduced pressure. In the triphasic extraction (TE), grapefruit seed powder (3 g) was extracted with a system of five solvents with different polarities (n-heptane/EtOAc/ACN/ButOH/H_2_O with proportion 22:14:29:8:27) [27]. The mixture was stirred for 14 h at 35 °C and decanted. The triphasic solvent system was filtered, separated by a separatory funnel and evaporated under reduced pressure. Limonin and nomilin content in the crude extract were determined by high-performance liquid chromatography (HPLC). The retention time was 15.68 min for limonin (**2**), 16.49 min for nomilin (**3**), and 17.35 min for obacunone (**1**). A linear correlation between limonin and nomilin concentration and area of peaks was obtained for the covered concentration ranges from 0.11 to 1.75 μg/mL, sustained by the regression coefficient (R^2^ = 0.9932 and 0.9310, respectively) (Figure 1 and Figure 2).

### 2.5. Virus Propagation 

Viral isolate BetaCov/Italy/CDG1/2020|EPI ISL 412973|2020-02-20 (GISAID accession ID: EPI_ISL_412973), obtained from a COVID-19 patient, was propagated by inoculation of 70–80% confluent Vero E6 cells in 75 cm^2^ cell culture flasks. Cells were observed for cytopathic effect (CPE) every 24 h. Stocks of SARS-CoV-2 virus were harvested at 72 h post infection, and supernatants were collected, clarified, aliquoted, and stored at −80 °C. Infectious virus titer was determined as Plaque Forming Units (PFU/mL). Virus propagation was conducted within biosafety level (BSL)-3 facilities at ISS (Rome, Italy).

### 2.6. Plaque Reduction Neutralization Test 

The method used for Plaque Reduction Neutralization Test (PRNT) was previously described [28]. The GSE compounds were resuspended in 30% dimethyl sulfoxide (DMSO), leading to a final concentration of 1 mg/mL that did not affect the growth of the cells in in vitro experiments. Then, serial 2-fold dilutions of GSE extracts (from 0.5 mg/mL to 0.015 mg/mL) were incubated with 80 PFU of SARS-CoV-2 at 4 °C overnight (~16 h). The mixtures were added in triplicates to confluent monolayers of Vero E6 cells, grown in 12-well plates and incubated at 37 °C in a humidified 5% CO_2_ atmosphere for 60 min. Then, 4 mL/well of a medium containing 2% Gum Tragacanth + MEM 2X supplemented with 2.5% of heat-inactivated FCS was added. Plates were left at 37 °C with 5% CO_2_. After 3 days, the overlay was removed, and the cell monolayers were washed with PBS to completely remove the overlay medium. Cells were stained with a crystal violet 1.5% alcoholic solution. The presence of SARS-CoV-2-infected cells was indicated by the formation of plaques. The half-maximal inhibitory concentration (IC_50_) was determined as the highest dilution of substance resulting in a 50% (PRNT50) reduction in plaques compared with the virus control. PRNT is a live neutralization assay that was conducted within biosafety level BSL-3 facilities at ISS.

### 2.7. Cell Cultures 

A549 (Lung epithelial) cells were cultured in T25 tissue culture flasks. RPMI Medium (Merck Life Science, Italy) was used, supplemented with 10% fetal bovine serum (FBS, Merck Life Science, Italy), 100 U/mL penicillin and 100 U/mL streptomycin and 2 mM L-glutamine in a humidified atmosphere of 95% air and 5% CO_2_ at 37 °C. Vero E6 (Cercopithecus aethiops-derived epithelial kidney, C1008ATCC CRL-1586) cells were grown in Minimum Essential Medium (MEM + GlutaMAX, Gibco) supplemented with 10% Fetal Calf Serum (FCS, Merck Life Science, Italy), 100 U/mL penicillin, 100 U/mL streptomycin, 1 mM sodium pyruvate, and 1% nonessential amino acids. 

### 2.8. Cell Viability and Cell Morphology

Cells were grown at a density of 2 × 10^4^ cell per well on 96-well plates in a final volume of 100 µL per well. After treatments, the cell viability was measured by MTS assay (Promega Italia S.r.l., Milan, Italy). MTS was utilized according to the manufacturer’s instructions. After cell treatments, the incubation was continued for 2 h at 37 °C in 5% CO_2_. The absorbance was read at 490 nm using the Microplate Reader GloMax fluorimeter (Promega Corporation, Italy). Cell viability was expressed as arbitrary units of absorbance, with the control group set to 1. For dose-dependent assay the A549 cells were treated with 0.01, 0.1, and 0.3 mg/mL of extracts and obacunone (**1**), limonin (**2**), nomilin (**3**) and narirutin (**4**) + naringin (**5**) for 24 h. For cytocompatibility analysis, Vero E6 cells were grown at a density of 1 × 10^4^ cell per well in 96-well plates and were treated for 24 h with semilog dilutions of **GSE3**, **GSE3_n-BuOH_**, obacunone (**1**), limonin (**2**) and nomilin (**3**) (from 0.5 mg to 0.125 mg). Then, 5 mL of tetrazolium salt XTT (Cell Proliferation Kit II, Roche, Mannheim, Germany) was added to the cells and 10% of Electron-Coupling Reagent was added, according to the manufacturer’s instructions. After incubating for 2 h at 37 °C, plates were read in a iMark™ Microplate Absorbance Reader spectrophotometer (Bio-Rad Laboratories Inc., Hercules, CA, USA) at 450 nm. After this incubation period, the formazan dye formed was quantified using a scanning multiwell spectrophotometer. The measured absorbance directly correlates to the number of viable cells. 

### 2.9. Analysis of Reactive Oxygen Species (ROS) 

For oxidative stress generation, 500 µM of TBH (*tert*-butyl hydroperoxide, Luperox^®^ TBH70X, Merck Life Science S.r.l., Italy) was used for 2 h, alone and in combination with grapefruit-seed-extract treatment. The control (Ctrl) groups received an equal volume of the medium. For the DCFH-DA assay (Abcam, Milan, Italy) extracts were utilized at 100 µg/mL for 2 h. The treated and control cells were analyzed by using microscopy (Axio Scope 2 microscope, Zeiss, Germany). At the end of the treatments, each sample was added to DCFH-DA (100 µM) and placed in the dark for 10 min at room temperature. After washing with PBS, cells were analyzed using a Microplate Reader GloMax fluorimeter (Promega Corporation, Milan, Italy) at the excitation wavelength of 475 nm and emission wavelength 530 nm for fluorescence intensity detection, and results were expressed as a percentage of the control group. Cell fluorescence was also visualized using the fluorescence microscope Zeiss Axio Scope 2 microscope (Carl Zeiss, Oberkochen, Germany).

### 2.10. Mitochondrial Membrane Potential Analysis

The mitochondrial transmembrane potential was measured by plating and treating the cells as mentioned above. After treatment with nomilin (**3**) at 0.1 mg/mL for 2 h, the cells were incubated for 30 min at 37 °C with 2 µM JC-1 red dye (5,5′,6,6′-tetrachloro-1,1′,3,3′-tetraethylbenzimidazolylcarbocyanine iodide) using the MitoProbe JC-1 assay kit (Thermo Fisher Scientific, Waltham, USA). Fluorescence emission shift of JC-1 from red (590 nm) to green (529 nm) was evaluated by the fluorescence microscope equipped with a 488 nm excitation laser using the Microplate Reader GloMax fluorimeter (Promega Corporation, Milan, Italy). 

### 2.11. Mitochondrial Morphology Analysis

For mitochondrial morphology analysis, A549 cells were treated with nomilin (**3**) at a concentration of 0.1 mg/mL for 2 h. After treatment, cellular mitochondria were stained using the MitoTracker Deep Red (Invitrogen, Carlsbad, CA, USA) dye (20 nM) for 15 min, washed twice with growth medium, and observed under the fluorescence microscope (488 nm excitation/590 nm emission). Original fluorescence images were converted to binary images. Mitochondrial shapes were obtained by visualizing the mitochondria outlines automatically drawn by the ImageJ software (Public Domain, BSD-2 license, National Institutes of Health, Bethesda, MD, USA). Morphometric mitochondria data, length and perimeter, were calculated from each mitochondrial outline (N 500).

### 2.12. Statistical Analysis

All experiments were repeated at least three times. Each experiment was performed in triplicate. The results are presented as a mean ± standard deviation (SD). The significance of the differences in the mean values was evaluated by using one-way analysis of variance (ANOVA) followed by Bonferroni’s post hoc test. Differences were considered significant when the value was *p* ≤ 0.05. SD of SARS-CoV-2 reduction in infectivity was calculated at different concentrations of each compound. In this study, a 95% confidence interval (CI) was considered.

## 3. Results

### 3.1. Chemical Isolation and Characterization 

#### 3.1.1. Isolation of Bioactive Compounds

A preliminary screening of the virucidal activity of extracts **GSE1** (hexane), **GSE2** (dichloromethane), and **GSE3** (ethanol/water 1:1) against SARS-CoV-2 showed that the most promising fraction was **GSE3** (see below for details). Thus, the efforts were focused on **GSE3**, the hydroalcoholic extract, to shed light on the molecules responsible for the activity. The extract was partitioned between water and n-butanol. The aqueous phase **GSE3_water_** contained mainly carbohydrates, as shown by ^1^H-NMR, and was inactive in biological assays. The alcoholic phase (**GSE3_n-BuOH_**) was purified by column chromatography on silica gel to obtain two main fractions. Further purifications were performed on **F1** and **F2**, as reported in scheme 1. The main isolated compounds belonged to the classes of limonoids and flavonoids. Obacunone (**1**) (0.2 mg/g DW), limonin (**2**) (1.4 mg/g DW), nomilin (**3**) (0.3 mg/g DW), narirutin (**4**) (0.6 mg/g DW) and naringin (**5**) (0.3 mg/g DW) were obtained as pure molecules (Figure 1 and Figure 2). Purity of all isolated compounds was proved to be ≥95% by means of HPLC, MS and NMR techniques (see Supplementary Materials).

#### 3.1.2. Comparison of Extraction Protocols

To optimize the recovery of the most promising compound, extraction methods alternative to SE were carried out: ethanol extraction (EtE), acetone/ethyl acetate extraction (AAE) and triphasic extraction (TE). The conditions and yields of all the extractions are reported in Table 1 and Table S1. Briefly, using SE, it was found that the grapefruit seeds contained 26% apolar compounds, such as fatty materials; while the DCM (2%) and EtOH/H_2_O (13%) extracts were composed of medium polar and polar compounds, such as aglycons and glycosides of limonoids and flavonoids. EtE and AAE were then investigated, as they required a single extraction step by green and sustainable solvents [28,29]. 

The total quantity of the main limonoids (limonin and nomilin) in percentage with respect to the dried matrix (DW) were 10% and 20% in EtE and AAE, respectively (calculated on the bases of the areas in HPLC chromatograms). The yield of EtE extraction (9.19 mg/g) was comparable to SE (DCM + EtOH/H_2_O, 9.49 mg/g). Conversely, the AAE extract was richer in limonoids (20.26 mg/g) than SE. 

Finally, we employed the triphasic method (TE). The triphasic extraction had the advantage of mixing five solvents with different polarities for a single extraction step, with a consequent reduction in process time and solvent consumption. The solvents used for the TE were chosen on the bases of the results reported in the paper by Gori et al. [27]. The yields of extraction of the apolar phase, intermediate phase and polar phase were 13%, 5% and 8%, respectively, and were composed largely of fatty materials, secondary metabolites and sugars, respectively, as showed by NMR spectra. This method was not convenient, as limonoids were distributed in all the phases.The results suggested that the most convenient and efficient extraction protocol was the one based on the use of green solvents acetone and ethyl acetate (AAE). 

### 3.2. Biological Activity

#### 3.2.1. Virucidal Activity on SARS-CoV-2

**Figure 3:**
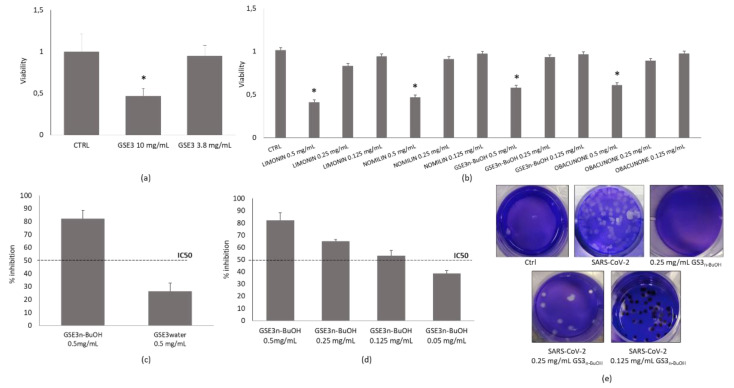
Virucidal effect against SARS-CoV-2. Cellular viability after exposure of Vero E6 cells to (**a**) **GSE3**; (**b**) **GSE3_n-BuOH_** extract and single components; (**c**) rate of viral of inhibition by **GSE3_n-BuOH_** and **GSE3_water_**; (**d**) rate of viral of inhibition by **GSE3_n-BuOH_** dilutions; (**e**) SARS-CoV-2 plaques after fixing and staining with crystal violet after 72 h. * *p* < 0.05 compared to control (CTRL) group.

After a preliminary screening of **GSE1**, **GSE2** and **GSE3**, we choose to focus on **GSE3** and its main components, which showed the higher virucidal activity when used to treat the virus before its inoculum on Vero E6 monolayers. Their capacity to neutralize the authentic virus SARS-CoV-2 was evaluated in vitro through PRNT50, tested in triplicates, and the concentration required to inhibit 50% of infection (IC_50_) was used as cut-off threshold of virucidal activity. A standard XTT viability assay was performed prior to the assessment of the virucidal activity of the hydroalcoholic extract **GSE3** to rule out the possibility that the compounds may have cytotoxic effects on the Vero E6 cells (Figure 3a,b). As mentioned above, we focused on **GSE3**, the hydroalcoholic extract and on the molecules contained therein (Figure 3c,d). A substantial difference was evidenced in the results obtained from tests on **GSE3_n-BuOH_** and **GSE3_water_**. As Figure 3 reports, the rate of inhibition of **GSE3_n-BuOH_** at 0.5 mg/mL settled at over 70% as result of its virucidal activity, compared to a similar concentration of **GSE3_water_**, which proved to be completely ineffective against SARS-CoV-2 (Figure 3c). The following dilution of 0.125 mg/mL is still effective, and the inhibitory effect fell below 50% at 0.05 mg/mL (Figure 3d), with an IC_50_ of 0.118 mg/mL. The visualization of viral plaques after staining with crystal violet shows that **GSE3_n-BuOH_** fraction is active against SARS-CoV-2, while it has no effect on cells after 72 h (Figure 3e).

**Figure 4:**
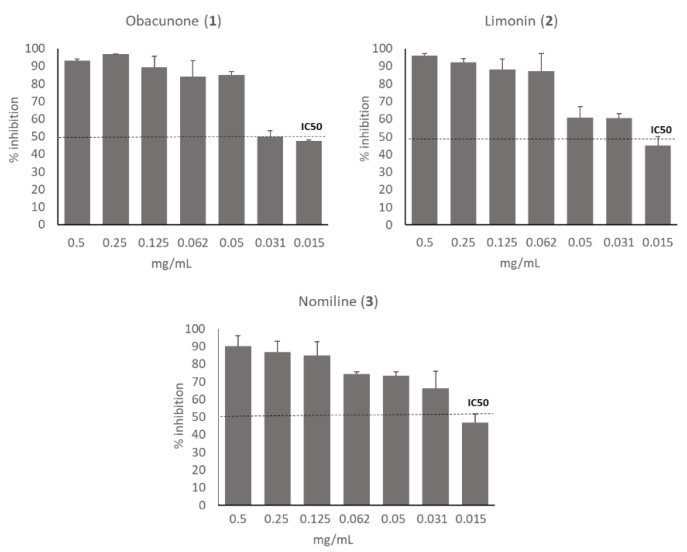
Dose-dependent inactivation of SARS-CoV-2 by obacunone (**1**), limonin (**2**), nomilin (**3**). Virucidal activity was assessed by calculating the rate of viral inhibition by PRNT on Vero E6 cells after ~16 h of contact of the substances with the virus. Half-maximal inhibitory concentration threshold, IC_50_, is shown.

Besides **GSE3_n-BuOH_** and **GSE3_water_**, the single limonoids obacunone (**1**), limonin (**2**), nomiline (**3**) of **F1** fraction, and **F2** fraction containing narirutin (**4**) + naringin (**5**) were also tested against the virus. Results demonstrated that the activity of **GSE3_n-BuOH_** was mainly due to limonoids. In fact, obacunone (**1**), limonin (**2**) and nomilin (**3**) were all effective against SARS-CoV-2, with IC_50_ ranging between 15 and 31 µg/mL, as shown in Figure 4. No inhibitory effect has been shown by the fraction **F2** containing narirutin (**4**) + naringin (**5**).

#### 3.2.2. Effect of Grapefruit Seed Extracts on Cell Viability 

**Figure 5:**
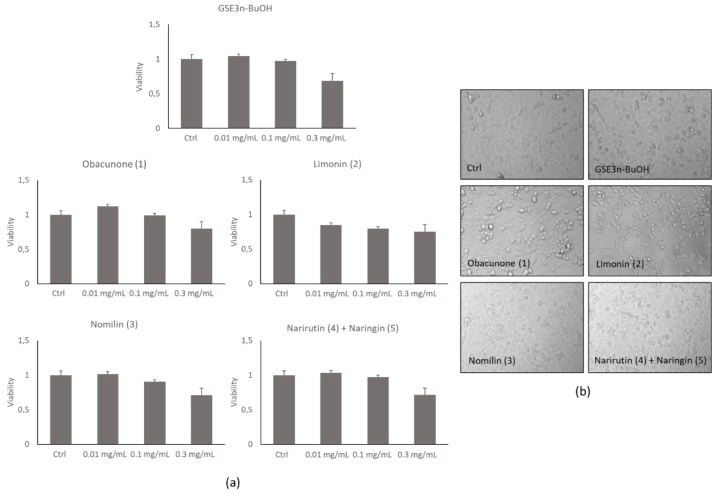
A549 cell viability and cell morphology of grapefruit seeds’ butanol extract (**GSE3_n-BuOH_**) and isolated pure compounds (obacunone (**1**), limonin (**2**), nomilin (**3**), narirutin (**4**) + naringin (**5**)). (**a**) Histograms of MTS cell viability assay. (**b**) Representative morphological images of untreated cells (Ctrl) and after 24 h from the addition of 0.1 mg/mL of the extracts and pure compounds.

**Figure 6:**
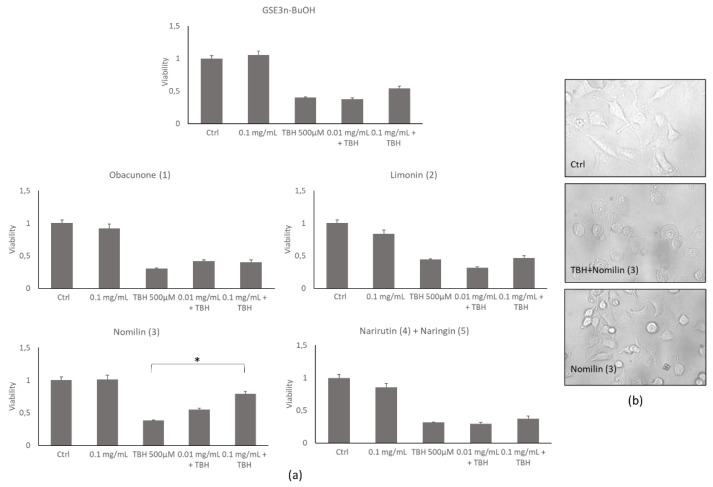
A549 cell viability and cell morphology of grapefruit seeds’ butanol extract (**GSE3_n-BuOH_**) and isolated pure compounds (obacunone (**1**), limonin (**2**), nomilin (**3**), narirutin (**4**) + naringin (**5**)). (**a**) Histograms of viability assay on untreated cells (Ctrl) or cells treated with TBH alone or with grapefruit seed extracts at increasing concentrations for 2 h. (**b**) Representative morphological images of untreated cells (Ctrl) or cells treated with TBH or cotreated with TBH and nomilin (**3**) at 0.1 mg/mL. * *p* < 0.05 compared to treated (TBH) group.

To evaluate the cytocompatibility of the grapefruit seed extracts, different concentrations of each extract or single pure molecules were added to lung epithelial cells—A459—for 24 h, and a MTS cell viability assay was performed. Figure 5a shows that no toxicity was detected at 0.01 and 0.1 mg/mL compared with the control. On the contrary, at 0.3 mg/mL, the extracts showed a decrease in cell viability, and for this reason this concentration was discarded for subsequent biological evaluations. This result was also confirmed by the morphological inspection of the cells treated at 0.1 mg/mL extract concentration (Figure 5b), where correct cell shape was observed, confirming the absence of any cell damage. The grapefruit seed extracts’ protective property was evaluated by treating cells with TBH 500 µM alone or in combination with increasing concentrations of the extracts and pure molecules after 2 h of incubation. As detected by MTS assay, only nomilin (**3**) was able to inhibit TBH-induced toxicity at 0.1 mg/mL (Figure 6a). This result was also confirmed by microscopic observation in which a significant recovery of the altered cell morphology was observed (Figure 6b). 

#### 3.2.3. Effects of Grapefruit Seed Extracts on ROS Production Induced by TBH Treatment 

**Figure 7:**
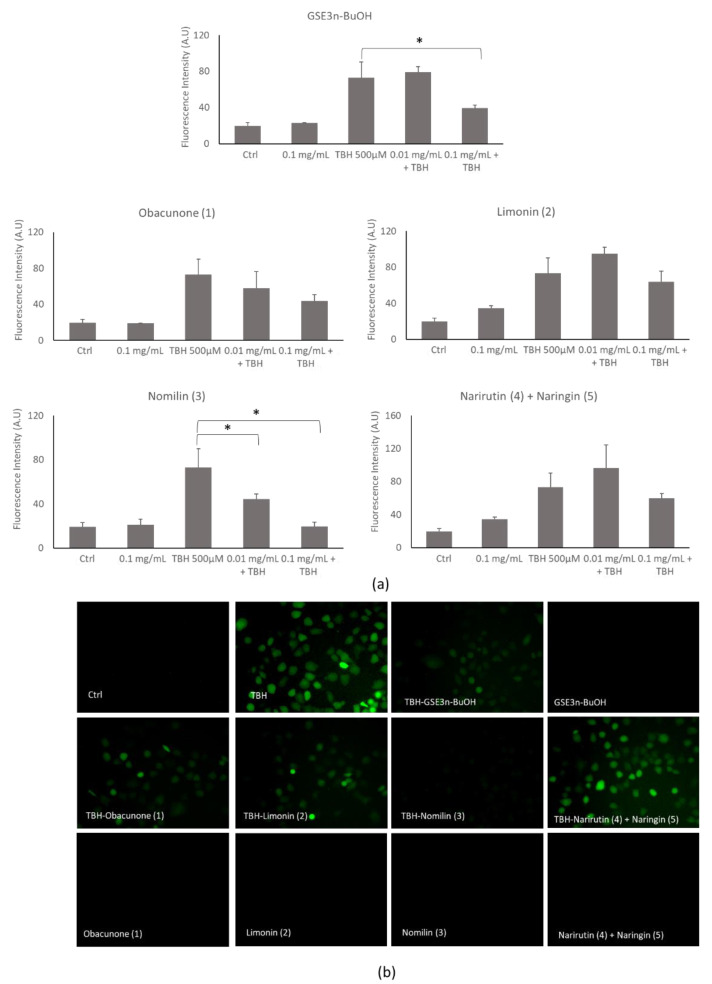
Reactive oxygen species analysis of grapefruit seeds’ butanol extract (**GSE3_n-BuOH_**) and isolated compounds (obacunone (**1**), limonin (**2**), nomilin (**3**), narirutin (**4**) + naringin (**5**)). (**a**) Histogram of ROS generation of untreated cells (Ctrl) or cells treated with 500 µM of TBH alone or in cotreatment with extracts at increasing concentrations for 2 h. (**b**) Representative cells’ fluorescence images of untreated cells (Ctrl) or cells treated with TBH or cotreated with TBH and grapefruit seed extracts at 0.1 mg/mL and treated with the extracts at 0.1 mg/mL each. * *p* < 0.05 compared to treated (TBH) group.

Grapefruit seed extracts’ antioxidant ability was analyzed by using a DCFH-DA assay. At 0.1 mg/mL all extracts presented a moderate decrease in TBH-induced ROS generation. Notably, **GSE3_n-BuOH_** significantly decreased TBH-induced ROS generation at 0.1 mg/mL. In particular, nomilin (**3**) significantly decreased TBH-induced ROS generation at 0.01 and 0.1 mg/mL (Figure 7a). The result was also confirmed by fluorescence microscope inspection (Figure 7b), in which untreated nomilin or nomilin (**3**) cotreated with TBH, did not show any fluorescence; in contrast, cells treated with TBH showed green fluorescence due to ROS generation. These results are in accordance with the effect of nomilin (**3**) on recovery the cellular viability when treated with TBH (Figure 7b). Based on these data, the nomilin (**3**) dose chosen for the subsequent protection experiment was 0.1 mg/mL.

#### 3.2.4. Effects of Nomilin (**3**) on Mitochondrial Membrane Potential Altered by TBH Treatment

**Figure 8:**
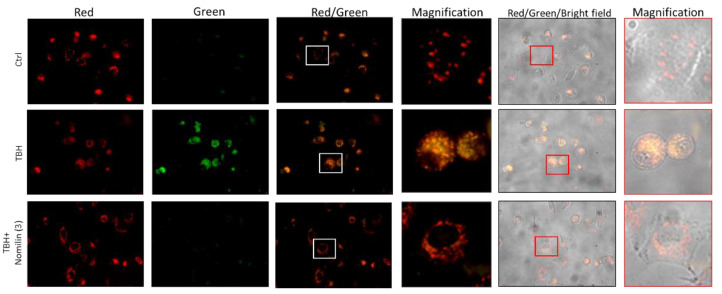
Mitochondrial membrane potential analysis of nomilin (**3**). Fluorescence microscope images of cells untreated (Ctrl) or treated with 500 µM of TBH alone or with nomilin (**3**) at 0.1 mg/mL for 2 h and submitted to JC-1 assay.

Excessive ROS production alters mitochondrial components, leading to mitochondrial dysfunction. Based on the experiments previously performed, the potential protective effect of nomilin (**3**) on mitochondrial dysfunction was evaluated. A549 cells treated with TBH at 500 µM for 2 h showed an intense green fluorescence, indicating that a high depolarization of the mitochondrial membrane occurred. Instead, cells cotreated with TBH and nomilin showed a higher red fluorescence emission similar to the untreated control (Ctrl) (Figure 8), clearly indicating a mitochondria-protective effect of the limonoid.

#### 3.2.5. Effects of Nomilin (**3**) on Mitochondrial Morphology

**Figure 9:**
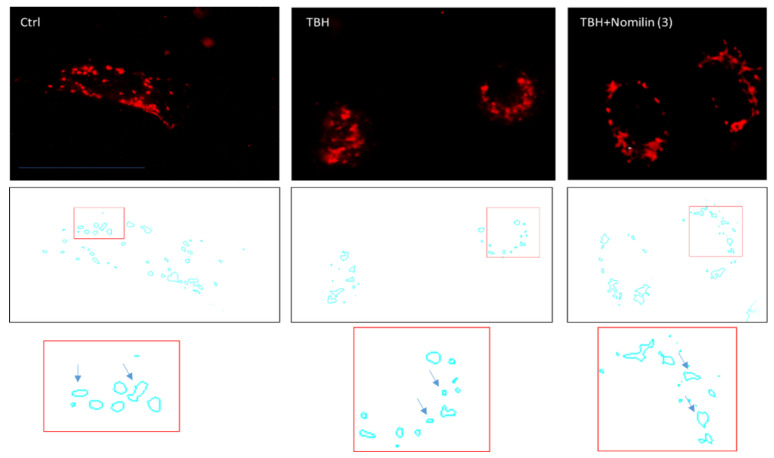
Mitochondrial morphology analysis. Representative fluorescence microscopy images of mitochondria stained with the MitoTracker Deep Red in untreated cells (Ctrl) or treated with 500 µM of TBH alone or in combination with nomilin (**3**) at 0.1 mg/mL for 2 h.

To analyze the mitochondria morphology, the A549 cells were stained with MitoTracker fluorescence dye. By microscopy fluorescence inspection, the control was characterized by an interconnected mitochondrial network with a tubular shape. Treatment of the cells with TBH resulted in a concomitant change in mitochondrial morphology from tubular networks to fragmented puncta (circular). When nomilin (**3**) was co-administered with the strong oxidizer TBH, the mitochondrial morphology was partially recovered (Figure 9).

## 4. Discussion 

The COVID-19 pandemic, induced by the worldwide spread of SARS-CoV-2, is easily identified for its clinical manifestations, which are consistent with respiratory diseases. While pulmonary alterations are the main manifestation of the disease, systemic problems are also significantly present, with complications including acute cerebrovascular events, brain disorders and other systemic diseases [1,2,3,4]. Countries have globally made a lot of effort to counteract the epidemic via appropriate public health strategies, developing drugs and vaccines against SARS-CoV-2 [30,31].

Despite all the efforts, COVID-19 remains a public health threat because of the complexity of mass vaccination programs, the lack of effective drugs and the emergence of new VOC (variants of concern). It has been recently found that a link between the risk of developing a severe COVID-19 infection and a high level of oxidative stress exists. Thus, a regulation of dysfunctional immune responses becomes as important as therapies inhibiting viral infection to block SARS-CoV-2 related pathologies. In this context, we focused our attention on natural compounds with the aim of finding molecules able to act through a two-pronged virucidal–antioxidant mechanism. In particular, we investigated the potential of grapefruit seed extracts (GSE) and of their main components, belonging to the class of limonoids. Limonoids are natural tetracyclic triterpenoids, widely distributed in the *Rutaceae* and *Meliaceae* families of the Citrus genus. These secondary metabolites can be found in several parts of plant, such as fruits, peels, root bark, roots, seeds, ect. [32] Limonoids are biosynthesized through isoprenoids pathway in the citrus seeds. [33] This class of molecules can be found in the free or glycosidic form. The first one is collected during the development of tissues, while the concentration of the second form increases during the maturation of seeds. Grapefruit seeds show the highest limonoids content, limonin and nomilin being the most abundant [34]. Various interesting biological activities of this class of compounds are reported, including antitumoral, anti-inflammatory, antiobesity, antihyperglycemic, antioxidative, anti-neurological-disease, anti-insecticidal, antibacterial and antiviral activities [35,36,37,38]. Interestingly, a recent work by Vardhan et al. evidenced the potential antiviral activity of limonoids on the basis of in silico ADMET and molecular docking studies on the inhibition of five SARS-CoV-2 protein targets [39]. Intrigued by these findings, we carried out a systematic evaluation of the antiviral activity against SARS-CoV-2 of GSE extracts and purified single molecules, obtained by the development of an efficient extraction protocol from the natural matrix. The results confirmed that the most active compounds belonged to the family of limonoids.

In particular, the components nomilin (**3**) and obacunone (**1**), showed the highest potency against the virus (IC**_50_** between 15 and 30 µg/mL) without any cytotoxic effects on the Vero E6 cells after 24 h. 

Given that viral infections are often associated with an acute oxidative stress influencing the pathogenesis of diseases, the use of antioxidants could aid the recovery of COVID-19 patients. 

Therefore, we analysed the antioxidant activity of the limonoids by in vitro experiments using the powerful oxidant TBH. We also investigated the cell viability, and ROS production induced by treatment of human lung epithelial cells with TBH in the presence of limonoids. Moreover, for the first time, we studied the effect of limonoids on mitochondrial dysfunction. Mitochondrial dysfunction driven by oxidative stress was blocked by treatment with nomilin (**3**), one of the isolated limonoids. Nomilin (**3**) inhibited oxidative-induced mitochondrial membrane damage, indicating that its radical-scavenging effect is also extended to mitochondrial ROS. 

## 5. Conclusions

In this work, we investigated the natural extract of grapefruit seeds as a source of active molecules to fight against SARS-CoV-2 infection by a dual approach, involving both virucidal and antioxidant activity. GSE extracts, in particular components of the extract belonging to the class of limonoids, are endowed with significant virucidal, antioxidant and mitoprotective activity. Obacunone (**1**), limonin (**2**) and nomilin (**3**) were all effective against SARS-CoV-2, with IC_50_ ranging between 15 and 31 µg/mL. In addition, nomilin (**3**) was also able to significantly decrease ROS production and oxidative-induced mitochondrial membrane damage at 0.1 mg/mL. Overall, our results demonstrate that GSE containing citrus limonoids could both directly target the virus and protect the hosting cell from ROS damage. More research will be needed to study the exact mechanism underpinning the virucidal activity of limonoids and to evaluate if GSE extracts and/or pure compounds could be considered for oral or nasal treatment of SARS-CoV-2 infection in the near future. 

In summary, targeting viruses and host-cell specific defense mechanisms at the same time represents an original and advantageous approach for developing novel virucidal agents. This strategy is characterized by a lower risk of resistance acquisition and possibly will give us the instruments to counteract the present disease as well as future pandemics. 

## Figures and Tables

**Table 1:** Content of main limonoids (limonin and nomilin) in the extracts.

Extraction Method	Extract	Total LM Content (mg/g DW ± SD)
SE	GSE1-Hexane	-
	GSE2-DCM	5.43 ± 1.60
	GSE3-Ethanol/H_2_O 1:1	4.05 ± 1.07
		**9.48**
EtE	EtOH	9.19 ± 1.34
AAE	Ac/AcOEt 1:1	20.26 ± 0.90
TE	Apolar phase	4.46 ± 0.22
	Intermediate phase	12.76 ± 1.25
	Polar phase	9.59 ± 1.04
		**26.81**

## Data Availability

The data presented in this study are available in this manuscript.

## Supplementary Materials

The following are available online at https://www.mdpi.com/article/10.3390/antiox10111794/s1, Table S1: condition and yields of all the extraction, NMR and MS characterization.
