# Supplementary material for: Antioxidant Activity of Citrus Limonoids and Investigation of Their Virucidal Potential against SARS-CoV-2 in Cellular Models
Source: Antioxidants (Basel). 2021 Nov 10;10(11):1794. doi: 10.3390/antiox10111794 (PMC8615075; doi:10.3390/antiox10111794)
Supplement: Supplementary file 1 [file antioxidants-10-01794-s001.zip › antioxidants-1402449-supplementary.pdf]

antioxidants

MDPI

Supplementary Materials:

Table S1. condition and yields of all the extraction.

Entry	Solvents	Temperature	Time	Seeds/solvent (g/mL)	Yield (%)
	Hexane				26
GSE1-3	DCM	r.t.	24h (x2)	20/100	2
	Etanol/H_2O 1/1				13
EtE	EtOH	r.t.	24h (x2)	3/10	10
AAE	Ac/AcOEt	r.t.	24h (x2)	3/10	26
					13 (Apolar phase)
TE	n-Heptane/AcOEt/ACN/ButOH/H_2O	35°C	7h (x2)	3/53.4	5 (Intermediate phase)
	(22:14:29:8:27)				8 (Polar phase)

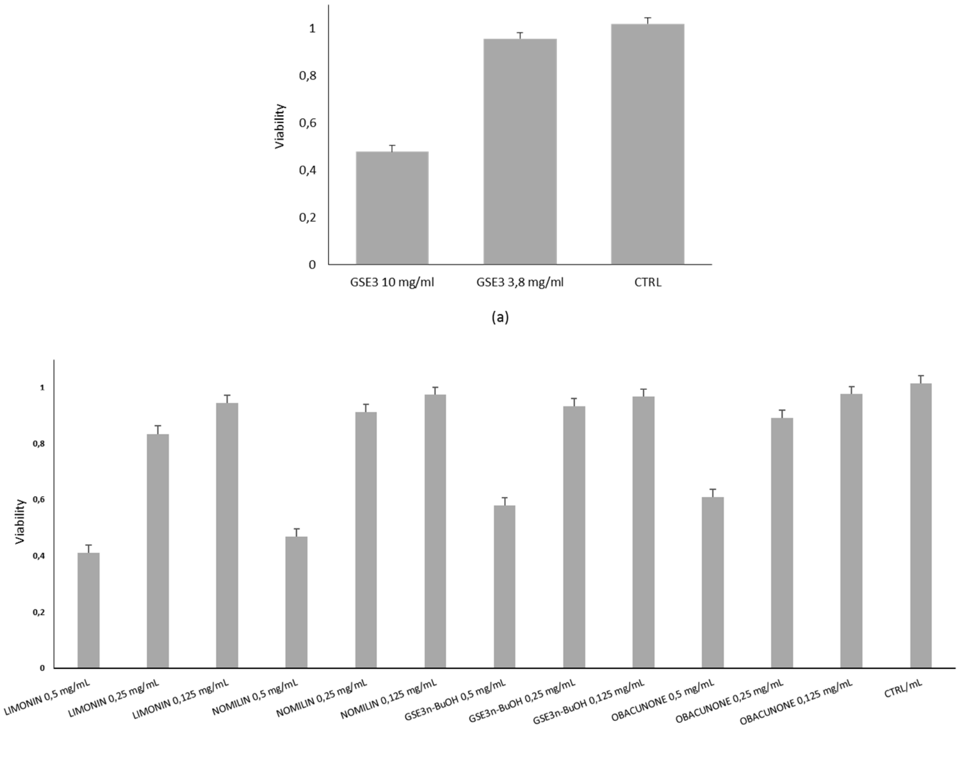

(b)

Figure S1. Cells viability by XTT assay. Level of cytotoxicity of GSE3 (a) and GSE3 main components (b).

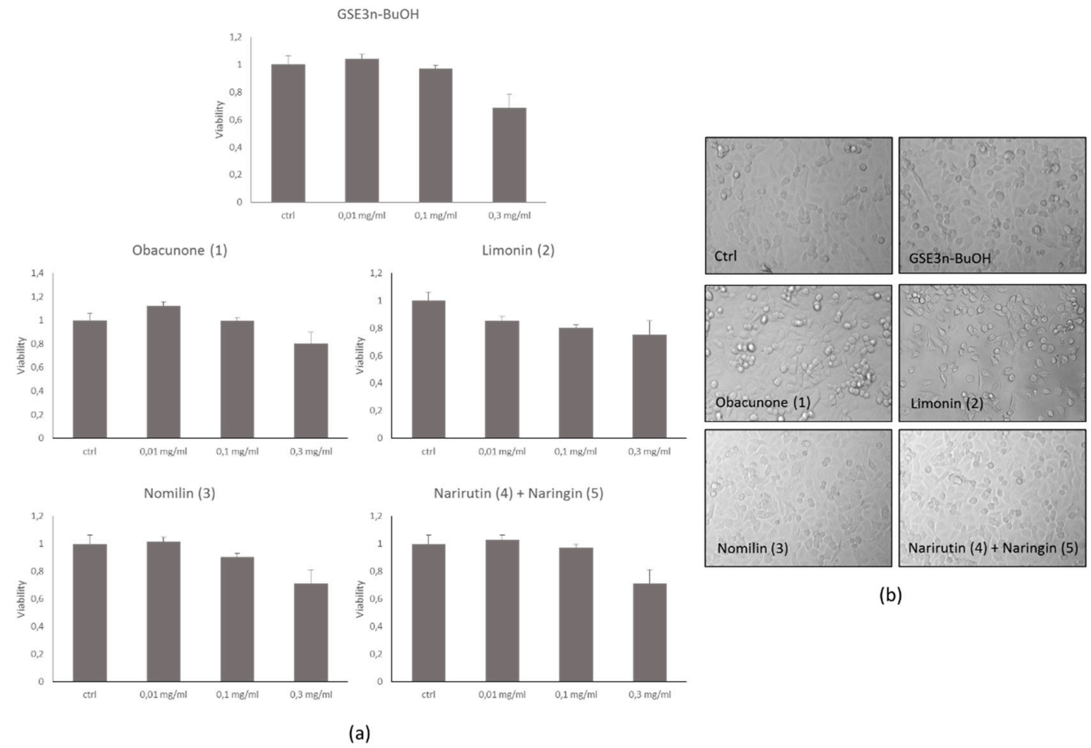

Figure S2. Cytotoxicity of the grapefruit seeds on A549 cells. (a) Histograms of MTS cell viability assay. (b) Representative morphological images of untreated cells (Ctrl) and after 24 hours from the addition of 0.1mg/mL of the extracts.

NMR and MS characterisation

The NMR experiments were performed on 600 MHz Bruker Advance instrument using deuterated chloroform (for limonoids) or methanol (flavonoid glicosides). The structures of isolated compounds were elucidated by 1D and 2D NMR experiments.

Limonin (2)[37]

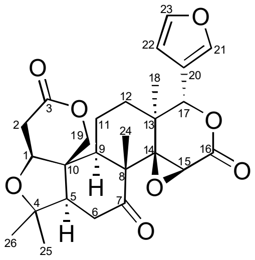

C	^{1}H-NMR	^{13}C-NMR	HMBC
**1**	4.03, brs	80.3	
**2**	2.85, t, *J*= 15.1 Hz	36.3	C10,C5, C1
	2.45, dd, *J*= 14.5, 3.0 Hz		
**3**	-	169.1	C19
**4**	-	79.1	
**5**	2.2, dd, *J*=15.6, 3.1 Hz	60.5	C25, C24,C9,C4
**6**	2.97, dd, *J*= 16, 3.1 Hz	35.6	C10,C4,C1
	2.65, dd, *J*= 14.4, 3.1 Hz		
**7**	-	206.1	
**8**	-	51.3	
**9**	2.54, dd, *J*= 12, 2.1 Hz	48.0	C24,C14,C4
**10**	-	45.9	
**11**	1.8, m	20.6	C12,C9,C13,C14
	1.5, m		
**12**	1.5, m	30.8	C13,C9
	1.78, m		
**13**	-	37.9	
**14**	-	65.6	
**15**	4.03, brs	53.8	C14, C9
**16**	-	166.6	
**17**	5.46, brs	77.8	C13,C14
**18**	1.06, s	17.6	
**19**	4.76, d, *J*= 13.1 Hz	65.3	C10,C4
	4.45, d, *J*=13.1 Hz		
**20**	-	119.9	C21,C22,C23,C17
**21**	7.40, brs	143.2	
**22**	6.33, brs	109.7	
**23**	7.41, brs	141.1	
**24**	1.28, s	31.0	C5,C4
**25**	1.16, s	18.8	
**26**	1.17, s	21.4	

HMRS *m/z* ($C_{26}H_{30}O_8$): 493.1830 (100%, M+ Na) 494.1863 (30%, M+1+Na)

Nomilin (3)[38]

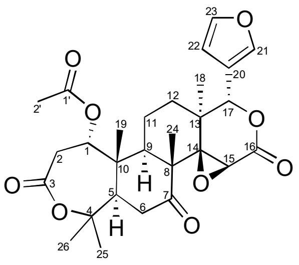

C	¹H-NMR	¹³C-NMR	HMBC
**1**	5, m	70.6	C19, C5, C3
**2**	3.20, d, *J*= 15.6 Hz	35.2	C10, C1, C3
	3.11, dd, *J*= 15.6, 7.3 Hz		
**3**	-	169.2	
**4**	-	84.3	
**5**	2.76, t, *J*= 15.2	50.9	C19, C9, C4, C7
**6**	2.57	38.7	C19, C24, C9, C4, C7
**7**	-	206.7	
**8**	-	52.8	
**9**	2.46, d, *J*= 10.3 Hz	44.3	C19, C 12, C15
**10**	-	44.1	
**11**	1.60, m	16.5	
**12**	1.77, m	31.9	
	1.10, m		
**13**	-	37.4	
**14**	-	65.4	
**15**	3.78, brs	53.3	C14, C16
**16**	-	166.7	
**17**	5.43, s	77.9	C12, C13, C14, C21, C20, C22
**18**	1.2, s	17.1	C9, C14, C7
**19**	1.3, s	17.0	C5, C1
**20**	-	120.0	
**21**	6.31, s	109.5	C20, C22
**22**	7.39, brs	143.2	C23, C20
**23**	7.39, brs	140.9	
**24**	1.16, s	20.8	
**25**	1.45, s	33.4	C4, C5
**26**	1.54, s	32.2	C4, C5
**CH_3CO:**			
**1′**	-	169.1	
**2′**	2.0, s	20.7	

HMRS *m/z* ($C_{28}H_{34}O_9$): 537.2101 (100%, M+Na) 538.3133 (30%, M+1+Na)

Obacunone (1)[39]

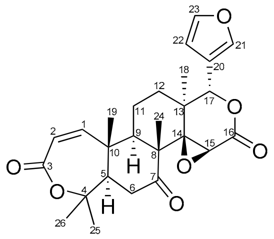

C	¹H-NMR	¹³C-NMR	HMBC
**1**	6.50, d, *J*= 11.8 Hz	156.7	C9, C3
**2**	5.96, d, J= 11.8 Hz	123.0	C10
**3**	-	166.9	
**4**	-	83.9	
**5**	2.59, dd, *J*= 14.0, 5.1 Hz	57.3	C25, C4
**6**	α- 2.29, dd, J= 14.0, 5.1 Hz<br>β- 2.98, t, J= 14.0 Hz	39.8	C5, C4, C7
**7**	-	207.0	
**8**	-	52.9	
**9**	2.14, dd, *J*= 9.0, 3.6 Hz	49.2	C12, C10
**10**	-	53.1	
**11 (α,**	1.88, m	19.4	
**β)**	1.6, m		
**12 (α,**	1.88, m	32.7	
**β)**	1.6, m		
**13**	-	37.4	
**14**	-	65.0	
**15**	3.66, s	53.8	C14, C16
**16**	-	166.6	
**17**	5.46, brs	77.8	C13, C14, C22, C20, C21
**18**	1.12, s	16.9	C17, C14
**19**	1.24, s	32.0	C7, C8
**20**	-	120.1	
**21**	7.41, brs	141.0	
**22**	6.36, brs	109.7	C20, C23
**23**	7.39, t, J= 1.7 Hz	143.1	C22, C20
**24 (30)**	1.50, s	21.1	
**25 (28)**	1.45, s	16.4	
**26 (29)**	1.50, s	26.7	C10, C9, C5, C4

HMRS *m/z* ($C_{26}H_{30}O_7$): 477.1888 (100%, M+ Na) 478.1920 (M+1+Na)

Narirutin (4)[40]

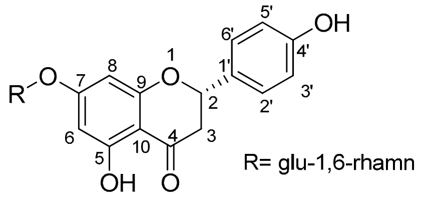

C	^{1}H-NMR	^{13}C-NMR
**2**	5.37, dd, J=12.80, 2.51 Hz	80.8
**3**	3.15, dd, J=12.80, 17.3 Hz	44.3
	2.74, dd, J=17.30, 2.85 Hz	
**4**		198.7
**5**		165.1
**6**	6.15, d, J=2.19 Hz	98.1
**7**		167.0
**8**	6.17, d, J= 2.19 Hz	97.2
**9**		159.2
**10**		105.1
**1′**		130.9
**2′6′**	7.31, d, J=8.40 Hz	129.3
**3′5′**	6.81, d, J= 8.40 Hz	116.5
**4′**		164.7
**Glu**		
**1**	5.09	101.3
**2 - 6**	3.38 – 3.86	74.8
		78.0
		71.4
		77.3
		67.5
**Rha**		
**1**	5.24	102.3
**2-5**	3.58 – 3.92	72.2
		72.5
		74.2
		69.9
**Me**	1.28	18.0

Naringin (5)[41]

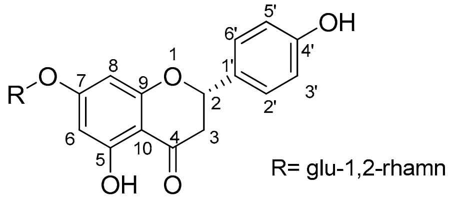

C	^{1}H-NMR	^{13}C-NMR
**2**	5.36,dd, J=12.68, 2.51 Hz	80.8
**3**	2.75, dd, J=12.80, 17.3 Hz	44.3
	3.89, dd, J=17.30, 2.85 Hz	
**4**		198.7
**5**		164.8
**6**	6.15, d, J=2.19 Hz	98.0
**7**		166.7
**8**	6.17, d, J= 2.19 Hz	96.9
**9**		159.3
**10**		105.0
**1′**		130.9
**2′6′**	7.31, d, J=8.02 Hz	129.3
**3′5′**	6.81, d, J= 8.02Hz	116.5
**4′**		165.1
**Glu**		
**1**	5.09	99.5
**2 - 6**	3.17 – 3.95	79.1
		78.2
		72.3
		74.1
		62.4
**Rha**		
**1**	5.24	102.7
**2 - 5**	3.42 – 3.66	71.4
		72.3
		79.3
		70.1
**Me**	1.20	18.3

HMRS *m/z* ($C_{27}H_{32}O_{14}$): 603.1697 (40%, M+Na) 604.1758 (100%, M+1+Na), 605.1809 (70%, M+2+Na)
